# Analysis of gene expression in monocytes of immunized pigs after infection with homologous or heterologous African swine fever virus

**DOI:** 10.3389/fvets.2022.936978

**Published:** 2022-08-12

**Authors:** Natalia Kholod, Andrey Koltsov, Galina Koltsova

**Affiliations:** Laboratory of Viral Genomics, Federal Research Center for Virology and Microbiology, Pokrov, Russia

**Keywords:** African swine fever virus, seroimmunotype, transcriptome, homologous ASFV strain, heterologous ASFV strain

## Abstract

African swine fever is a deadly disease of pigs caused by the large DNA virus (ASFV). Despite intensive research, little is known about the molecular mechanisms of ASFV pathogenesis. Transcriptome analysis of host and viral genes in infected macrophages revealed changes in expression of genes involved in various biological processes, including immune response, inflammatory response and apoptosis. To understand the mechanisms of virus pathogenesis, we used transcriptome analysis to identify the differences in gene expression between peripheral blood monocytes (PBMCs) isolated from pigs immunized with attenuated Congo ASFV strain (KK262), and then infected *in vitro* with virulent homologous Congo strain (K49) or heterologous Mozambique strain (M78). We found that overexpression of IFN-γ was detected only in cells infected with M78, although the expression of interferon-stimulated genes was increased in both types of cells. In addition, up-regulation of pro-inflammatory cytokines and chemokines was found in PBMCs infected with the heterologous strain M78, in contrast to the cells infected with K49. These data may indicate the beginning of an early immune response in cells infected with a heterologous, but not homologous strain. Transcriptome analysis revealed down-regulation of genes involved in endocytosis and phagocytosis in cells infected with the K49 strain, but not in PBMCs infected with M78. On the contrary, we detected activation of endoplasmic reticulum stress response genes in cells infected with a homologous strain, but not in cells infected with a heterologous strain. This study is the first attempt to determine the differences in the response to ASF infection between homologous and heterologous strains at the cellular level. Our results showed that not only genes of the immune response, but also genes involved in endocytosis and cellular stress response may be important for the formation of cross-protective immunity. This data may be useful for vaccine development or testing of candidate vaccines.

## Introduction

African swine fever (ASF) is an acute viral hemorrhagic disease of domestic pigs and wild boars with a mortality rate close to 100% (1). After being imported from East Africa to Georgia, the ASFV has been circulating in Eastern Europe since 2007, in the European Union since 2014 and in Asia since 2018 (2). The spread of the disease outside Africa has become a global threat with enormous economic losses for pig-raising countries (3). It is well-known that ASF is caused by a large icosahedral cytoplasmic virus belonging to the *Asfarviridae* family (4, 5). The ASFV genome is a double-stranded DNA molecule of 170–194 kb in size, which contains 150–167 open reading frames (ORFs), depending on the virus strains (4, 5). Based on the sequence of the B646L gene encoding the p72 capsid protein, 24 ASFV genotypes were identified (6, 7). In addition, eight ASF serogroups (SG) have been identified based on serological typing in the hemadsorption inhibition (HAI) reaction, although there are probably more serogroups (8, 9).

There is no vaccine against ASF, although it has been reported that animals immunized with an attenuated ASFV strain were protected from challenge by a homologous virulent virus of the same serogroup (10–13). However, such protection was not observed in the case of challenge by a heterologous virulent virus of another serotype (14, 15).

Protective immunity against ASF remains insufficiently defined. Antibodies neutralizing ASF have been described (16–19), but they alone are not enough to protect animals (17, 20). Apparently, the cellular immune response also plays an important role in protecting pigs from ASF, and cytokines and chemokines, including interferons, are among the key factors in this immune response. It has been shown that the secretion of IFNγ by lymphocytes correlates with the degree of protection of animals immunized with low-virulent isolates from infection by a homologous virulent strain (21–24). However, the level of IFNγ secretion did not correlate with the formation of cross-protection against a heterologous strain (22, 25–27). It is also still unknown whether cytokine levels correlate with cross-protection against ASF in pigs. In addition, it is possible that not only cytokines and chemokines are involved in the molecular mechanisms of the formation of a protective immune response or, conversely, the development of pathological processes. Understanding these mechanisms is crucial for the development of ASF vaccines.

“Omics” technologies, such as genomics, transcriptomics, proteomics, metabolomics and others, represent a powerful and high-performance approach for the global analysis of biological samples. The application of these methods has made a significant contribution to our understanding of various human and animal diseases and, for example, the interaction of viruses and their host. In the ASFV study, methods of proteomics and transcriptomics of virus-infected cells were used to analyze the expression of viral genes during infection (28–30). As a result, a complete map of ASFV genes expressed in mammalian cells from the early to late stages of infection was compiled, as well as a catalog of proteins that make up virus particles (30, 31). In addition, DNA microarray and RNA sequencing (RNA-seq) techniques have been used to study differences in the transcription of host genes of ASFV infected cells. Thus, cDNA microarrays were used to study the expression of host genes in cells infected with either wild-type or attenuated strains of ASFV (32, 33). Using RNA-seq to analyze gene expression during ASFV infection may be useful to study the mechanism describing the host's response to ASFV infection. Recently, RNA-Seq technology has been applied for transcriptome study of whole blood or tissues isolated from pigs infected with either low pathogenic ASFV-OURT 88/3 or high-virulent ASFV-GRG strains (34). There are also several transcriptome studies that have investigated how host macrophages respond to infection with the virulent Georgia 2007/1 (35, 36), Chinese CN/GS/2018 (37) or Pig/Heilongjiang/2018 (Pig/HLJ/18) ASFV isolates (38). Transcriptome analysis identified differently expressed genes of various biological processes, including inflammatory and innate immune responses, cytokine and chemokine signaling, antiviral response, apoptotic and metabolic pathways (32–40). Thus, studies of gene expression profiles in macrophages infected with ASFV Georgia 2007 revealed increased production of pro-inflammatory TNF cytokines, as well as down-regulation of anti-inflammatory cytokine IL10, that may cause an excessive inflammatory tissue response (35). In addition up-regulation of chemokines and interferons, as well as calcium binding proteins S100 has been reported (36). Another study of the transcriptome of macrophages infected with ASFV Georgia 2007 (ASFVCN/GS/2018) showed differential expression of antiviral factors, as well as chemokines and apoptotic genes, but the immune and inflammatory response was very limited (37). Transcriptomic analysis of macrophages infected with ASFV Pig/Heilongjiang/2018 (Pig/HLJ/18) at different time points showed strong inhibition of genes associated with host immunity, while strengthening chemokine-mediated signaling pathways and impaired regulation of host metabolism, which contributed to virus replication and pathogenesis (38). It is worth noting that due to the different conditions of the experiments, the results of different studies are somewhat difficult to compare with each other.

In this study, we characterized changes in gene expression in peripheral blood monocytes (PBMC) of animals immunized with a live attenuated ASFV strain KK262 (Congo-a, Genotype I, Serogroup 2) after infection of these cells with homologous virulent strain K49 (Congo-v, Genotype I, Serogroup 2), or heterologous virulent strain M78 (Mozambique-v, Genotype V, Serogroup 3).

## Materials and methods

### Cell cultures and viruses

Attenuated ASFV Congo-a strain (KK262, Genotype I, Serogroup 2), parent virulent ASFV Congo-v strain (K49, Genotype I, Serogroup 2) and virulent ASFV Mozambique-v strain (M78, Genotype V, Serogroup 3) were received from the reference collection of the Federal Research Centre for Virology and Microbiology, Russia. Strain K49 was originally isolated in 1949 from a domestic pig (*Sus scrofa domesticus*) in Katanga province of the Democratic Republic of the Congo. The ASFV strain KK262 is a derivative of the highly virulent strain K49, obtained as a result of 50 consecutive passages in pig kidney cell lines (SPEV) and 262 passages in pig bone marrow cell culture (41). Strain M78 was originally isolated in Mozambique and transferred to the Federal Research Center for Virology and Microbiology in 1978, but the exact date of the outbreak is unknown.

Peripheral blood mononuclear cells (PBMCs) were prepared from defibrinated blood using the Lymphocyte separation media (Gibco). Cells (1.3 × 10^6^ cells/well) were cultured in 96-well plates (Corning) in RPMI 1640 medium supplemented with 30% (v/v) plasma, 10% (v/v) fetal bovine serum (Gibco) and antifungal antibiotic (Gibco) at 37°C with 5% CO_2_ for 24 h. The adhering cells were rinsed with the same PBMC medium and used in assays after 48 h.

Virus titration was performed on 96-well plates by visualizing of CPE in PBMCS of pigs. Titers were expressed as mean of tissue culture infectious dose (TCID50) according to the Reed–Muench method (42).

### ASFV infection of PBMCs

Peripheral blood mononuclear cells (PBMCs) were prepared from animals immunized with ASFV Congo-a in the experiment described earlier (43). Briefly, pigs were infected intramuscularly with 10^6^ TCID50 of Congo-a virus. At 21 day post-infection (dpi), the animals were boosted with the same dose of the same virus. Three weeks later (42 dpi.), PBMCs were isolated from defibrinated blood using the Lymphocyte separation media (Gibco). The cells were resuspended in RPMI 1640 medium supplemented with 30% (v/v) plasma, 10% (v/v) fetal bovine serum (Gibco) and antimycotic-antibiotic (Gibco). The washed cells (1 × 10^6^cells/well) were seeded into 48 well plates and incubated for 2 h at 37°C with 5% CO_2_. Then the cells were inoculated with two different virulent viruses with the multiplicity of infection of 1 (MOI = 1). Five hours after inoculation with the virus, PBMCs were washed once with sterile PBS and used to isolate total RNA.

To identify the genome of the ASFV, PCR of the B646L gene was performed in accordance with the protocol published by King et al. (44). PCR of the β-actin gene was used as endogenous control. PCR reactions were carried out on a CFX96TM thermal cycler (Bio-Rad, Hercules, CA, USA).

### RNA extraction and sequencing

The total RNA was isolated with Trisol LS reagent and PureLink RNA micro Kit (Invitrogen) according to manufacture instruction. The analysis of the quality of the obtained RNA was carried out on the Bioanalyzer 2100 using RNA 6000 Nano Kit (Agilent Technologies) according to the recommendations of the manufacturer. PolyA RNA was purified with Dynabeads^®^ mRNA Purification Kit (Ambion). Illumina library was made from polyA NEBNext^®^ Ultra™ II RNA Library Prep (NEB) according to manual. Sequencing was performed on HiSeq 1500 system with 50 bp read length. At least 10 millions of reads were generated for each sample. Reads were aligned with the porcine genome using STAR aligner and differentially expressed transcripts were count by DESeq2.0 (45).

### Statistical and bioinformatic analyses

Log2fold changes in signal intensity were applied in statistical analysis to identify differentially expressed genes (DEGs). The *P*-values were calculated using R software DESeq2.0. To account for multiple testing, the *p*-values were adjusted using the Benjamini and Hochberg method, and the false discovery rate (FDR)-corrected *P*-value were calculated. Differences in gene expression with a FDR-value of 0.05 or less and an expression difference of 50% or more were considered as DEGs. The genes up- or down-regulated were expressed as positive and negative values (fold), respectively. The identified DEGs were compared with human reference genes. The bioinformatics program (DAVID Bioinformatics Resources 6.8) was used to identify biological pathways (GOTERM_BP_DIRECT, REACTOME_PATHWAY and KEGG_PATHWAY) for significantly different DEGs.

## Results

### Host gene expression changes

Peripheral blood mononuclear cells isolated from pigs immunized with attenuated ASFV strain Congo-a (KK262) were infected *in vitro* with the virulent homologous strain Congo-v (K49) or the heterologous strain Mozambique-v (M78). Infection of cells with the ASF virus was confirmed by real-time PCR using primers specific to the ASFV B646L gene. PCR results showed the presence of almost the same amount of viral genome in infected samples (Supplementary Table 1).

To investigate the difference in gene expression in these infected cells compared to non-infected ones, total RNA was isolated at 5 hpi and sequenced. A total of 24,358 genes were analyzed, and it was found that the expression of 2,255 genes changed significantly in cells infected with K49 strain compared to non-infected cells (Table 1). When the cells were infected with the M78 strain, the expression of only 774 genes was different. Comparison of expression profiles between cells infected with homologous K49 or heterologous M78 viruses showed that 1,918 genes were expressed differently. Among them, the expression of 1,235 genes was increased in the case of cells infected with strain K49, and the expression of 544 genes in the case of cells infected with strain M78. The number of down-regulated genes was 1,020 and 230 for cells infected with K49 or M78 strains, respectively. When comparing the gene expression profiles of cells infected with K49 vs. cells infected with M78, 920 genes were up-regulated and 998 genes were down-regulated, correspondently (Table 1).

**Table 1:** The number of differently expressed genes (DEG) in PBMCs infected with ASFV strains K49 or M78 (FDR ≤ 0.05).

**Differently expressed genes**	K49 vs. Neg^*^	M78 vs. Neg^*^	**K49 vs. M78**
Total	2,255	774	1,918
Significantly up-regulated	1,235	544	920
Significantly down-regulated	1,020	230	998

* * Neg, non-infected macrophages*.

### Pathway analysis

Analysis of differently expressed genes homologous to human ones showed that 10 biological pathways were significantly up-regulated in PBMCs infected with both K49 and M78 viruses (Table 2). First of all, these pathways are involved in biological processes related to the cellular response to pathogens, as well as interferon alpha/beta and cytokine signaling. The largest number of up-regulated genes was found in the pathways of response to viral infection: 42 genes for K49 and 36 genes for M78 infected cells. These pathways manly consist of interferon-induced/stimulated genes (Table 2). In addition, a number of up-regulated genes of the protein polyubiquitination pathway were found in both types of infected PBMCs. This pathway is important for the regulation of many processes such as protein translation and degradation, endocytic trafficking and inflammation (46, 47).

**Table 2:** Biological pathways with up-regulated genes in PBMCs infected with both K49 and M78 strains (FDR ≤ 0.05).

**Biological process**	**Pathway N**	**Pathway**	**K49**		**M78**	
			**Count**	**padj**	**Count**	**padj**
Response to virus	GO:0051607	Defense response to virus	42	3.3 × 10^−10^	36	4.2 × 10^−17^
	GO:0045071	Negative regulation of viral genome replication	14	3.1 × 10^−4^	12	3.6 × 10^−6^
	R-HSA-1810476	RIP-mediated NFkB activation via ZBP1	8	4.0 × 10^−2^	6	2.6 × 10^−2^
Interferon alpha/beta signaling	GO:0060337, R-HSA-909733	Type I interferon signaling pathway	23	4.7 × 10^−7^	23	3.4 × 10^−13^
	R-HSA-168928	DDX58/IFIH1-mediated induction of interferon-alpha/beta	9	2.4 × 10^−3^	8	1.1 × 10^−4^
	GO:0032480, R-HSA-936440	Negative regulation of type I interferon production	11	2.4 × 10^−3^	9	2.1 × 10^−4^
	R-HSA-933542	TRAF6 mediated NF-kB activation	9	1.7 × 10^−2^	7	7.1 × 10^−3^
Cytokine Signaling	GO:0060397, R-HSA-982772	Growth hormone receptor signaling	9	1.7 × 10^−2^	10	1.5 × 10^−5^
	GO:0007259	JAK-STAT cascade	10	2.0 × 10^−2^	10	4.0 × 10^−5^
Protein ubiquitination	GO:0000209	Pprotein polyubiquitination	25	8.8 × 10^−2^	15	4.0 × 10^−2^

Comparison of the pathways with DEGs in cells infected with homologous or heterologous strains revealed that the most significant difference was associated with the unfolded protein response (17 genes were up-regulated in PBMCs infected with K49) (Table 3). In addition, genes involved in protein modification by phosphorylation (46 genes) and ubiquitination (29–32 genes), as well as genes involved in protein translation, chromatin organization and mRNA decay pathways were up-regulated in cells infected with K49 strain. In PBMC infected with the M78 strain, it was found that only clathrin-mediated endocytosis pathway was significantly up-regulated compared to cells infected with K49 strain.

**Table 3:** Biological pathways with differently expressed genes in PBMCs infected with strain K49 compared to infected with strain M78 (FDR ≤ 0.05).

**Biological process**	**Pathway N**	**Pathway**	**Count**	**padj**
**Up-regulated in K49 over M78**				
Response to cellular stress	GO:0036499, R-HSA-380994	PERK-mediated unfolded protein response	7	4.0 × 10^−3^
	GO:1990440	Positive regulation of transcription in response to ER stress	6	3.9 × 10^−2^
	GO:0030968	Endoplasmic reticulum unfolded protein response	17	8.1 × 10^−7^
	GO:0042149	Cellular response to glucose starvation	8	8.2 × 10^−2^
Chromatin organization	GO:0000123	Histone acetyltransferase complex	9	1.1 × 10^−3^
Protein phsophorylation	GO:0006468	Protein phosphorylation	46	1.7 × 10^−2^
Translation	GO:0006418, R-HSA-379716	tRNA aminoacylation for protein translation	9	2.0 × 10^−2^
Ubiquitination	GO:0004842	Ubiquitin-protein transferase activity	32	8.9 × 10^−2^
	GO:0016874	Protein ligase activity	29	4.8 × 10^−2^
mRNA decay	GO:1900153	Deadenylation-dependent mRNA decay	6	7.4 × 10^−3^
**Up-regulated in M78 over K49**				
Endocytosis	GO:0035615	Clathrin adaptor activity	6	2.1 × 10^−2^
	GO:0030122	AP-2 adaptor complex	5	6.5 × 10^−2^

### Analysis of differently expressed genes

#### Interferon-stimulated genes

There are 25 interferon-stimulated genes that were over-expressed in cells infected with both K49 and M78 strains, although it was found that the expression of interferon gamma alone was increased 10-fold only in PBMCs infected with the heterologous strain M78 (Table 4). It is interesting to note that 10 genes stimulated by interferon were up-regulated to a greater extent (over 1.5-fold difference) in cells infected with homologous strain K49 than with the heterologous strain M78. Among them, the expression of three genes (IFI 6, IFIT 5, and OAS 2) was more than 2 times higher in cells infected with the K49 strain compared to cells infected with M78. The expression of only three genes (DDX60, SOCS1, and SOCS3) was 2 times higher in cells infected with the heterologous strain M78 compared to PBMCs infected with the homologous strain K49.

**Table 4:** Differential expression of interferon-stimulated genes in PBMCs infected with ASFV strains K49 or M78 (FDR ≤ 0.05).

**Gene**	**Base Mean**	**k49 vs. Neg**		**M78 vs. Neg**	
		**Fold**	**padj**	**Fold**	**padj**
IFNG	84	1.5	5.9 × 10^−1^	16.3	6.3 × 10^−24^
BST2	429	6.0	3.6 × 10^−42^	4.7	1.8 × 10^−32^
DDX58	718	8.6	5.5 × 10^−104^	5.6	6.9 × 10^−61^
DDX60	153	3.9	7.3 × 10^−9^	5.7	1.8 × 10^−19^
DHX58	304	8.6	5.5 × 10^−104^	5.6	6.9 × 10^−61^
GBP2	354	4.3	1.3 × 10^−35^	3,5	4.9 × 10^−26^
IFI44L	817	3.0	2.5 × 10^−38^	2,7	2.7 × 10^−34^
IFI6	579	5.0	7.7 × 10^−60^	2,3	2.2 × 10^−14^
IFIH1	762	3.6	1.1 × 10^−53^	2,6	9.9 × 10^−27^
IFIT1	1,069	6.5	8.3 × 10^−80^	4,3	3.8 × 10^−61^
IFIT2	285	8.1	1.5 × 10^−31^	5,6	2.1 × 10^−7^
IFIT5	161	8.2	8.7 × 10^−26^	3,5	1.5 × 10^−6^
IFITM3	561	4.0	4.4 × 10^−44^	3,5	3.9 × 10^−45^
IRF1	3,313	6.9	1.1 × 10^−212^	5,4	4.2 × 10^−226^
ISG15	1,710	4.9	1.3 × 10^−86^	3,1	2.4 × 10^−70^
ISG20	113	12.1	4.5 × 10^−26^	12,6	1.6 × 10^−29^
MX1	2,659	5.9	6.9 × 10^−144^	4,4	3.4 × 10^−37^
MX2	2,605	9.3	5.6 × 10^−240^	6,1	4.4 × 10^−164^
OAS2	2,143	8.6	3.2 × 10^−40^	3,6	2.2 × 10^−13^
RSAD2	580	8.3	2.7 × 10^−76^	5,1	3.4 × 10^−50^
SOCS1	169	4.9	6.4 × 10^−22^	9,3	2.1 × 10^−64^
SOCS3	2,525	2.4	5.9 × 10^−53^	4,8	1.6 × 10^−243^
UBE2L6	1,051	4.2	5.1 × 10^−76^	3,3	4.4 × 10^−59^

#### Cytokines

RNA-seq analysis of PBMCs infected with the ASFV M78 strain revealed up-regulation of 9 different cytokine signaling pathways compared to uninfected cells (Supplementary Table 2). Changes in these pathways were not detected in PBMCs infected with ASFV K49 strain (Supplementary Table 3). However, a comparison of cytokine signaling pathways between PBMCs infected with heterologous strain M78 or homologous strain K49 showed only up-regulation of the JAK-STAT cascade and grow hormone receptor signaling in both cell types (Table 2). No other differences in the expression of cytokine signaling pathways were found (Table 3).

Analysis of the expression of individual cytokine genes showed that only a few of them were expressed differently in the compared cells (Table 5). Over-expression of 3 interleukin genes (IL6, IL10, and IL33) was detected in cells infected with the heterologous strain M78, and among them the transcription of IL6 was 16.2 times higher compared to cells infected with the homologous strain K49. The greatest difference in expression was found for the IL33 gene (121.5 times), but this difference appeared due to the fact that, on the one hand, the expression of the IL10 gene in cells infected with the M78 strain was 13.4 times higher compared to uninfected cells, and on the other hand, it was 10 times lower in cells infected with the K49 strain compared to uninfected cells (Table 5).

**Table 5:** Differential expression of cytokine genes in PBMCs infected with ASFV strains K49 or M78 (FDR ≤ 0.05).

**Gene**	**Base Mean**	**M78 vs. K49**		**M78 vs. Neg**		**K49 vs. Neg**	
		**Fold**	**padj**	**Fold**	**padj**	**Fold**	**padj**
IL10	670	4.3	9.6 × 10^−48^	5.8	6.3 × 10^−77^	1.3	6.6 × 10^−2^
IL33	544	121.5	2.1 × 10^−71^	13.4	1.4 × 10^−143^	0.1	3.7 × 10^−9^
IL6	86	16.2	8.2 × 10^−19^	44.6	2.9 × 10^−22^	2.7	2.8 × 10^−1^
TNF	366	4.9	5.1 × 10^−35^	7.0	1.9 × 10^−57^	1.4	1.1 × 10^−1^

TNFa was found to be significantly up-regulated only in cells infected with the M78 strain, but the expression of most TNFa-induced genes did not change in either K49-infected or M78-infected cells compared to uninfected PBMCs (data not shown).

#### Chemokines

Only 8 differently expressed chemokines were found in cells infected with the heterologous strain M78 compared to cells infected with the strain K49 (Table 6). Four of them (CXCL10, CXCL11 CXCL2, and CXCL8) were up-regulated in both cell types with a greater degree in PBMCs infected with the M78 strain. Three other chemokines (CCL11, CCL2, and CCL3L1) were significantly down-regulated in cells infected with the homologous strain K49 (1.4–10 times), and at the same time up-regulated in cells infected with the strain M78 (2.5–10 times). Thus, the difference in the expression of these chemokines ranged from 10 to 32 times. The greatest difference expression was observed for CXCL9 gene, since transcripts were not detected in uninfected or K49-infected cells, and more than 100 copies were detected in PBMCs infected with the heterologous M78 strain.

**Table 6:** Differential expression of chemokine genes in PBMCs infected with ASFV strains K49 or M78 (FDR ≤ 0.05).

**Gene**	**Base Mean**	**M78 vs. K49**		**M78 vs. Neg**		**K49 vs. Neg**	
		**Fold**	**padj**	**Fold**	**padj**	**Fold**	**padj**
CCL11	659	10.4	1.4 × 10^−104^	7.5	8.1 × 10^−111^	0.7	9.8 × 10^−2^
CCL2	1,826	19.1	6.8 × 10^−286^	2.5	5.6 × 10^−59^	0.1	5.2 × 10^−101^
CCL3L1	4,916	31.9	2.6 × 10^−138^	8.6	5.1 × 10^−73^	0.3	4.1 × 10^−39^
CXCL10	1,169	2.5	3.9 × 10^−4^	69.6	2.1 × 10^−50^	27.3	5.6 × 10^−36^
CXCL2	28,325	3.1	9.6 × 10^−15^	4.3	1.0 × 10^−52^	1.4	7.8 × 10^−3^
CXCL8	38,980	2.2	2.7 × 10^−12^	7.1	1.2 × 10^−106^	3.3	2.1 × 10^−106^
CXCL9	114	114.0	4.1 × 10^−4^	114.0	1.6 × 10^−8^	ND^*^	ND^*^
AMCF-II	17,678	13.34	1.0 × 10^−23^	3.55	1.7 × 10^−232^	0.27	5.7 × 10^−7^

* *ND, not determined*.

Alveolar macrophage chemotactic factor 2 (AMCF-II) was also expressed differently in cells infected with the M78 strain compared to cells infected with the K49 strain (over expression by 13.3 times). This difference was due to the fact that AMCF-II transcription was reduced by 3.3 times in PBMCs infected with strain K49 and at the same time increased by 3.5 times in cells infected with strain M78.

#### Genes involved in endocytosis and phagocytosis pathways

Pathway analysis also showed that significant differences in gene expression were associated with endocytosis/phagocytosis pathway (Table 3 and Supplementary Table 3). Among these pathways, most genes were down-regulated in cells infected with homologous strain K49, but not up-regulated in cells infected with heterologous strain M78 (Table 7). The expression of only 5 genes (DAB2, HIP1, LYN PIK3CB, and VAV1) was increased by about 2 times in cells infected with the M78 strain compared to uninfected cells. The greatest difference in expression between PBMCs infected with M78 or K49 was found for the DAB2 and ITGB8 genes, by 11 and 13 times, respectively. If for DAB2, there was a 2-fold increase in gene expression in cells infected with the M78 strain and, at the same time, a 5-fold decrease in cells infected with the K49 strain. Then, for the ITGB8 gene, a decrease in the number of transcripts was found in both cell types, by 2 times in cells infected with M78, and by 25 times in PBMCs infected with the K49 strains.

**Table 7:** Differential expression of genes involved in endocytosis and phagocytosis in PBMCs infected with ASFV strains K49 or M78 (FDR ≤ 0.05).

**Pathway**	**Gene**	**Base mean**	**M78 vs. K49**		**M78 vs. Neg**		**K49 vs. Neg**	
			**Fold**	**padj**	**Fold**	**padj**	**Fold**	**padj**
Endocytosis	AP2A2	409	1.6	1.7 × 10^−5^	0.7	4.5 × 10^−4^	0.5	5.2 × 10^−17^
	DAB2	66	10.9	2.5 × 10^−13^	2.3	7.3 × 10^−5^	0.2	2.1 × 10^−4^
	HIP1	60	4.3	4.5 × 10^−7^	2.0	6.4 × 10^−3^	0.5	3.8 × 10^−2^
	PICALM	1,399	1.9	7.4 × 10^−20^	1.5	4.1 × 10^−12^	0.8	2.3 × 10^−3^
Phagocytosis	ACTB	13,333	1.9	3.3 × 10^−47^	1.1	6.6 × 10^−2^	0.6	1.8 × 10^−37^
	ACTG1	6,318	2.3	6.6 × 10^−62^	1.3	2.2 × 10^−9^	0.6	1.1 × 10^−30^
	ARPC5	1,779	2.2	3.4 × 10^−32^	1.1	7.0 × 10^−2^	0.5	1.2 × 10^−22^
	FGR	173	2.6	2.6 × 10^−8^	1.3	7.6 × 10^−2^	0.5	5.2 × 10^−4^
	HCK	347	2.3	8.5 × 10^−12^	1.3	1.7 × 10^−2^	0.6	1.6 × 10^−5^
	LYN	1,703	2.5	1.8 × 10^−43^	1.9	3.1 × 10^−33^	0.8	4.3 × 10^−4^
	PIK3CB	151	2.8	3.9 × 10^−9^	2.6	2.7 × 10^−10^	1.0	8.9 × 10^−1^
	VAV1	1,496	2.3	1.1 × 10^−34^	1.8	1.7 × 10^−23^	0.8	1.8 × 10^−3^

#### Genes involved in cellular stress response

Another pathway that was up-regulated in PBMCs infected with homologous strain K49, but not heterologous strain M78, was associated with a response to cellular stress (Table 3). Among the genes of this pathway, transcription of the Sgk1 protein kinase gene alone was repressed by 1.5-fold in cells infected with the M78 strain and was increased by 2 times in cells infected with the K49 strain (Table 8). The expression of other genes did not change significantly in cells infected with the M78 strain, but up-regulated from 1.5 to 6.5 times in cells infected with the K49 strain. The greatest difference in expression was found for the gene of asparagine synthetase ASNS, which is an important response factor to many cellular stimuli and various type of stress.

**Table 8:** Differential expression of genes involved in cellular stress response in PBMCs infected with ASFV strains K49 or M78 (FDR ≤ 0.05).

**Gene**	**Base mean**	**M78 vs. K49**		**M78 vs. Neg**		**K49 vs. Neg**	
		**Fold**	**padj**	**Fold**	**padj**	**Fold**	**padj**
ASNS	589	0.2	2.4 × 10^−66^	1.3	3.2 × 10^−1^	6.5	1.9 × 10^−77^
ATF4	6,933	0.4	4.5 × 10^−63^	1.1	1.9 × 10^−2^	2.7	2.8 × 10^−86^
ATF6	732	0.6	2.5 × 10^−8^	0.9	7.9 × 10^−1^	1.5	5.5 × 10^−7^
EIF2AK3	678	0.4	1.1 × 10^−24^	0.9	8.7 × 10^−1^	2.4	1.1 × 10^−23^
XBP1	1,092	0.6	2.9 × 10^−10^	1.5	1.1 × 10^−6^	2.4	3.9 × 10^−30^
SGK1	2,234	0.3	4.9 × 10^−80^	0.64	2.3 × 10^−13^	2.0	5.0 × 10^−36^

## Discussion

Previously, RNA-seq technology was used to study transcriptomes of macrophages infected with virulent or attenuated ASFV strains during the viral infection cycle. These studies revealed differences in the transcription of host genes in infected cells at different time points (35–38). It has been shown that immune and inflammation-related pathways are activated immediately during viral infection, and then repressed by proteins produced by ASFV (36, 37). In our study, we focused on comparative RNA-seq analysis of PBMCs of animals immunized with an attenuated ASFV strain, and then stimulated *in vitro* by two serologically different virulent strains to expand our understanding of the early determinants of response to homologous and heterologous infection.

We found that the total number of differently expressed genes in PBMCs infected with homologous strain K49 is 3 times higher compared to the same cells infected with the heterologous strain M78 (Table 1). Perhaps this is due to the fact that the cells had previously encountered an attenuated virus of the same strain and their reaction to it turned out to be faster. Analysis of biological pathways showed that for both types of infected PBMCs, a large number of up-regulated genes belong to pathways involved in the response to viral infection and interferon and cytokine signaling (Table 2 and Supplementary Tables 2, 3), which is consistent with previous reports (35, 37, 38). Interferons (IFNs) are key molecules of the immune system in response to viral infection. The production of the main interferons (IFN-α and IFN-γ) initiates a signaling cascade leading to the regulation of transcription of multiple genes in viral infection (48–50). In addition, low doses of recombinant porcine IFNs (IFN-α and IFN-γ) significantly inhibited the replication of ASFV both *in vitro* and *in vivo* (51). It is believed that type I IFNs plays an important role in inhibiting ASFV replication, but the role of IFN-γ is controversial. For example, it has been reported that inoculation of pigs with a non-virulent strain OURT88/3 induces a high number of IFN-γ-producing lymphocytes *in vivo* (21). However, in other studies, IFN-γ secretion was not detected in pigs during infection with virulent ASFV SY18 (52). It was also reported that the expression of IFN-γ did not change at the early stage of infection of macrophages with ASFV Georgia 2007 strain, but doubled at the late stage of infection (35). Our data showed that cells infected with the homologous K49 strain did not overexpress any interferons, but cells infected with the heterologous M78 strain showed a 16-fold increase in IFN-γ production. Therefore, we could expect an increase in the transcription of interferon-stimulated genes (ISGs) only for PBMCs infected with a heterologous strain. Unexpectedly, we found a significant increase in the production of interferon-induced genes in both types of cells, and this increase was even higher in cells infected with K49 strain compared to cells infected with M78 strain. These results indicate that IFN-independent regulation of ISGs may occur during ASFV infection, especially when the influx of pro-inflammatory IFNs may be harmful to the host. In studies of other viral infections, it is becoming increasingly obvious that a number of well-known ISGs can be regulated directly by infection without the need for IFN production (53–55). Interferon-stimulated genes are involved in various cellular processes in response to viral infection, such as induction of apoptosis and regulation of immune responses [for review see (56–58)]. However, many questions about their mechanism of action remain and require further study. Only three interferon-stimulated genes (SOCS1, SOCS3, and DDX60) were more up-regulated in PBMCs infected with M78 strain compared to cells infected with K49 strain. Induction of SOCS1 has already been shown at an early stage of macrophage infection with the virulent Georgia 2007 strain (35). Cytokine signaling suppressor proteins (SOCS) play a crucial role in the complex control of the inflammatory response through their action on various signaling pathways (59). However, many viruses have developed mechanisms that induce the expression of the host SOCS gene, which contributes to the survival of the virus (60, 61). Accumulated data indicate that SOCS1 and/or SOCS3 may have different effects on the severity of viral diseases depending on the type of cells (62).

Cytokines and chemokines play a key role in all aspects of immune responses, and therefore the study of their expression during ASFV infection has been the goal of many studies. We found that only four of cytokines were expressed differently in cells infected with the heterologous strain M78, compared with cells infected with the homologous strain K49. An increase in the level of pro-inflammatory cytokines IL-6 and TNF was previously shown both in macrophages infected with ASFV (37, 63) and in the serum of infected pigs (52, 64). Stimulation of the expression of these pro-inflammatory cytokines together with IFN-γ may reflect the initiation of an early immune response in PBMCs infected with a heterologous strain but not homologous. In contrast, IL-10 is the main immune-regulatory cytokine that has anti-inflammatory functions, limiting excessive tissue destruction caused by inflammation (65). Although it has also been reported that IL-10 may exhibit an IFN-γ-mediated pro-inflammatory effect in the presence of an ongoing serious inflammatory process (66). Increased expression of IL-10 was detected at the terminal stage of ASFV infection, which may be a sign of irreversible immune status, foreshadowing a fatal outcome (52). In addition, the lack of long-term protection in domestic pigs immunized with attenuated ASFV isolates correlated with an increased levels of IL-10 (67). We observed an increase in IL-10 expression at an early stage of infection in PBMCs infected with a heterologous strain, which may indicate a serious inflammatory process compared to cells infected with a homologous strain. It is known that IL-33 is a member of the IL-1 family of pro-inflammatory cytokines, which is constitutively expressed in normal tissues (68, 69). It acts as an alarm (“alarmin”), released when cells or tissues are damaged, to alert immune cells. It was shown that in macrophages IL-33 consistently triggered early expression of pro-inflammatory genes and activation of macrophages to eliminate inflammation and repair tissues (70). Moreover, activation of immune response cells led to a rapid decrease in IL-33 levels, which promoted wound healing (71). Recently, it has been proposed to use IL-33 as an adjuvant to enhance innate and humoral immune responses in the development of an effective vaccine for animals (70). We found a strong decrease in IL-33 expression in cells infected with K49 strain, despite the absence of pro-inflammatory cytokines. Conversely, a significant induction of IL-33 synthesis was observed in cells infected with the M78 strain, even in the presence of pro-inflammatory cytokines. Our data suggest that host cells or ASFV may regulate their cytokine response to infection by altering the expression of IL-33 to avoid a cytokine storm and an inflammation. But the exact mechanism needs to be investigated further.

Chemokines are chemotactic cytokines necessary for cell migration and, acting together with cytokines, contribute to the formation of a protective immune response [for review see (72)]. Many studies have reported increased expression of pro-inflammatory chemokines CXCL2, CXCL8, and CXCL10 at different stages of ASFV infection (34–38). Thus, a high level of CXCL10 expression was observed in PBMCs of pigs infected with virulent ASFV isolates (34), as well as in macrophages infected *in vitro* with Georgia 2007 (35) and GS/2018 strains (37). However, another study showed that pigs infected with highly virulent Benin97/1 virus had a sharp increase in CCL2, but only a slight increase in CXCL10 (73). And the data of the transcriptome analysis of macrophages infected with the virulent Pig/HLJ/18 ASFV isolate showed that CCL3L1 and CXCL8 were highly up-regulated (38). We found an increased level of expression of chemokines CXCL2, CXCL8 and CXCL10 in PBMCs infected with both viruses, but to a higher degree in cells infected with strain M78 compared with cells infected with strain K49. Three others chemokines CCL11, CCL2, and CCL3L1 were up-regulated in cells infected with the heterologous strain M78, but at the same time down-regulated in cells infected with the homologous strain K49. Chemokines CCL2 and CCL3L1 are known to recruit classical pro-inflammatory monocytes to the site of injury, whereas CCL11 attracts eosinophils, which are a marker of allergic inflammatory reactions (74). Transcripts of the chemokine CXCL9 were not found in uninfected cells or in cells infected with the homologous strain K49, but significant amounts of it were found in cells infected with the heterologous strain M78. The role of this chemokine in the inflammation process is unclear, but it is known that it binds to the CXCR3 receptor and attracts activated T-cells (75). Interestingly, the expression of chemokines attracting pro-inflammatory monocytes and activated T-cells (CCL2, CCL3L1, and CXCL9) was suppressed in cells infected with a homologous ASFV strain, which may reflect the inhibition of the inflammatory process. Increased expression of the alveolar macrophage chemotactic factor 2 (AMCF-II) was detected in cells infected with strain M78, and at the same time its down-regulation was shown in PBMCs infected with strain K49. It has been shown that AMCF-II, which is a pig-specific member of the IL-8 family, has chemotactic activity against neutrophils, but its role in the inflammatory and immune response remains unclear (76). Thus, during infection with a heterologous strain, up-regulation of chemokines attracting various types of immune cells was observed and additional studies are needed to determine the role of these chemokines in the pathogenesis of ASF.

Transcriptome analysis revealed significant differences in the gene expression of endocytosis and phagocytosis pathways between cells infected with heterologous or homologous ASFV strains. However, this difference occurred due to the fact that the expression of genes of these pathways is slightly increased in cells infected with M78 strain, and at the same time it is reduced in cells infected with K49 strain. Several mechanisms of ASF virus penetration into host cells have been proposed. Although the viral receptor(s) are still unknown, there is support for the mechanisms of virus penetration, such as clathrin-mediated dynamin-dependent endocytosis (77, 78), and there is also evidence that phagocytosis (79) and macropinocytosis are used (80, 81). We found that among the clathrin-mediated endocytosis (CME) genes, the difference in the expression of DAB2 was the most pronounced. Interestingly, DAB2 is not only involved in endocytosis, but is a negative regulator of the immune response and inflammation (82, 83). It has been shown that DAB2 is usually highly expressed in macrophages and sharply decreases after activation of cells by pathogenes (82). This decrease may affect the ability of macrophages to phagocyte, process and present antigens to T cells. Consequently, the down-regulation of DAB2 in cells infected with a homologous ASFV strain may also reflect the inhibition of the inflammatory process.

Macropinocytosis is an actin-dependent non-selective uptake process of large extracellular particles. It has been reported that macropinocytosis is a constitutive process in swine macrophages and is not induced by ASFV, although it is used by the virus to enter the cell (81). Indeed, we did not observe up-regulation of genes of phagocytosis/macropinocytosis in PBMCs infected with a heterologous strain, but rather a decrease in the expression of these genes in cells infected with a homologous strain. It is unclear whether this is due to the specific regulation of phagocytosis/macropinocytosis genes or to general changes in the translation of cellular proteins in response to viral infection. The only gene of the macropinocytosis pathway whose expression was significantly enhanced in cells infected with M78 strain, is PIK3CB, encoding the catalytic subunit of p110ß tyrosine kinase PI3Kß. Macropinocytosis has been shown to be highly dependent on PI3Kß, and loss of expression or activity of this kinase blocks macropinocytosis in the early stages (84). It is possible that an increase in PI3Kß expression in cells infected with a heterologous strain actually increases micropinocytosis, facilitating the penetration of the virus. Or maybe this kinase is involved in another process in the response to a viral infection that has not yet been investigated.

A large number of ASFV proteins are synthesized and accumulated in the endoplasmic reticulum (ER) of infected cells. There is more and more evidence that this process causes ER stress and an unfolded protein response (UPR) of the host cell (85–88). It has been reported that the ASFV protein K205R is capable of causing ER stress and the unfolded protein response by activating the transcription factor ATF6, the ER to nucleus and PERK signaling pathways (85). Another study showed that the vero-adapted ASFV isolate BA71V is able to induce ATF4, but not PERK signaling in infected vero cells (87). Surprisingly, we found no differences in the expression of genes involved in the cellular stress response in PBMCs infected with the heterologous strain M78 (except for the down-regulation of SGK1 kinase). However, we observed up-regulation of transcription factors ATF4 and ATF6, as well as PERK in cells infected with homologous strain K49. The most significant differences between cells infected with K49 or M78 strains were observed in the expression of the SGK1 kinase and asparagine synthetase (ASNS) genes. Serine/threonine-protein kinase SGK1 is involved in the regulation of a wide range of cellular processes and can be considered as last line of defense against cell damage [for review see (89)]. Asparagine synthetase (ASNS) is a critical enzyme involved in the synthesis of asparagine. It was demonstrated that knockdown of ASNS resulted in an early restriction in human cytomegalovirus (HCMV) replication, but had little effect on replication of herpes simplex virus 1 (HSV-1) or influenza A virus (IAV) (90). These data suggest that the loss of ASNS may have a specific effect on HCMV replication, and not be a consequence of simple depletion of asparagines in the cell. Activation of the ER-stress response of cells infected with a homologous strain at an early stage of infection may provide an advantage for cell survival. Conversely, delaying this response may provide benefits for virus replication when infected with a heterologous strain.

This work is the first attempt to study the differences in the response to ASFV infection between homologous and heterologous strains at the cellular level. Our results illustrate that not only cytokines, chemokines and interferon-stimulated genes may be important for the formation of cross-protective immunity, but also genes involved in endocytosis and response to cellular stress may play a significant role in the resistance of cells to viral infection. Our data may be useful for vaccine development or testing of candidate vaccines.

## Data availability statement

The data presented in the study are deposited in the https://www.ncbi.nlm.nih.gov/sra/PRJNA860828.

## Ethics statement

The animal study was reviewed and approved by the Research Ethics Committee of the Federal Research Center of Virology and Microbiology, Russia (No 3/2021).

## Author contributions

NK, AK, and GK contributed to the conception and methodology, design of the study, and executed the study. NK wrote the first draft of the manuscript. AK and GK wrote sections of the manuscript. NK and GK reviewed and edited the manuscript. GK exercise project administration. All authors have read and agreed to the published version of the manuscript.

## Funding

This research was funded by the Russian Science Foundation (grant no. 20-76-10030).

## Conflict of interest

The authors declare that the research was conducted in the absence of any commercial or financial relationships that could be construed as a potential conflict of interest.

## Publisher's note

All claims expressed in this article are solely those of the authors and do not necessarily represent those of their affiliated organizations, or those of the publisher, the editors and the reviewers. Any product that may be evaluated in this article, or claim that may be made by its manufacturer, is not guaranteed or endorsed by the publisher.
