# Supplementary material for: Analysis of gene expression in monocytes of immunized pigs after infection with homologous or heterologous African swine fever virus
Source: Front Vet Sci. 2022 Aug 12;9:936978. doi: 10.3389/fvets.2022.936978 (PMC9411669; doi:10.3389/fvets.2022.936978)
Supplement: Supplementary file 1 [file Data_Sheet_1.docx]

Supplementary Material

# Supplementary Tables

# Table 1. Real-time PCR results for ASFV B646L and β-actin genes.

| № | Sample | PCR Result | |
| --- | --- | --- | --- |
|  |  | ASFV B646L gene  Mean Ct | β-actin gene  Mean Ct |
| 1 | Congo-v (K49) | 18,55 | 18,1 |
| 2 | Mozambique-v (M78) | 18,43 | 18,1 |
| 3 | Neg (Control) | Neg | 17,7 |

Table 2. Biological pathways with up-regulated genes in PBMCs infected with strain M78 compared to uninfected cells (FDR ≤0.05)

| Biological process | Pathway number | Pathway | Count | padj |
| --- | --- | --- | --- | --- |
| Cytokine Signaling | R-HSA-912694 | Regulation of IFNA signaling | 6 | 5.7x10^-2^ |
|  | GO:0071347 | Cellular response to interleukin-1 | 13 | 1.5x10^-4^ |
|  | R-HSA-512988 | Interleukin-3, Interleukin-5 and GM-CSF signaling | 7 | 7.1x10^-3^ |
|  | R-HSA-912526 | Interleukin receptor SHC signaling | 6 | 6.1x10^-2^ |
|  | R-HSA-1169408 | ISG15 antiviral mechanism | 11 | 1.1x10^-2^ |
|  | R-HSA-5668541 | TNFR2 non-canonical NF-kB pathway | 9 | 7.3x10^-2^ |
|  | GO:0043032 | Positive regulation of macrophage activation | 5 | 1.3x10^-2^ |
|  | R-HSA-1059683 | Interleukin-6-mediated signaling pathway | 6 | 1.5x10^-3^ |
|  | GO:0060334,  R-HSA-877312 | Regulation of IFNG signaling | 7 | 3.7x10^-4^ |
| Signaling by chemokins | GO:0070098,  R-HSA-380108 | Chemokine receptors bind chemokines | 14 | 2.8x10^-5^ |
|  | GO:0008009 | Chemokine activity | 11 | 4.7x10^-4^ |
|  | GO:0006935 | Chemotaxis | 15 | 1.3x10^-3^ |
|  | GO:0002548 | Monocyte chemotaxis | 8 | 1.0x10^-2^ |
|  | GO:0002690 | Ppositive regulation of leukocyte chemotaxis | 5 | 4.7x10^-2^ |
| Interferon gamma signaling | GO:0071346 | Cellular response to interferon-gamma | 8 | 4.3x10^-2^ |
|  | GO:0032689 | Negative regulation of interferon-gamma production | 8 | 1.1x10^-3^ |
| Response to cellular stress | GO:0071356 | cellular response to tumor necrosis factor | 14 | 1.8x10^-3^ |
| Signal transduction | R-HSA-392451 | G beta:gamma signalling through PI3Kgamma | 8 | 4.5x10^-2^ |
|  | R-HSA-1433557 | Signaling by SCF-KIT | 10 | 3.7x10^-4^ |
|  | R-HSA-2586552 | Signaling by Leptin | 5 | 1.6x10^-2^ |
| Apoptosis | GO:2001244,  R-HSA-111453 | Positive regulation of intrinsic apoptotic signaling pathway | 9 | 4.0x10^-4^ |
|  | GO:0090200 | Positive regulation of release of cytochrome c from mitochondria | 7 | 7.6x10^-3^ |
| Tyrosine phosporylation | GO:0042517 | Positive regulation of tyrosine phosphorylation of Stat3 protein | 9 | 1.1x10^-3^ |
|  | GO:0018108 | Peptidyl-tyrosine phosphorylation | 15 | 1.0x10^-2^ |
|  | GO:0031234 | Extrinsic component of cytoplasmic side of plasma membrane | 9 | 2.5x10^-2^ |
|  | GO:0004713 | Pprotein tyrosine kinase activity | 13 | 8.6x10^-2^ |
| Platelet activation, signaling and aggregation | R-HSA-114604 | GPVI-mediated activation cascade | 10 | 7.1x10^-3^ |

# Table 3. Biological pathways with differently expressed genes in PBMCs infected with strain K49 compared to uninfected cells (FDR ≤0.1)

| Biological process | Pathway number | Pathway | Count | padj |
| --- | --- | --- | --- | --- |
| **Up-regulated** | | | | |
| Response to cellular stress | GO:0036499,  R-HSA-380994 | PERK-mediated unfolded protein response | 9 | 4.7x10^-5^ |
|  | GO:1990440 | response to endoplasmic reticulum stress | 7 | 7.6x10^-3^ |
| Ubiquitination | GO:0004842 | ubiquitin-protein transferase activity | 44 | 3.4x10^-3^ |
| Circadian clock | GO:0032922 | circadian regulation of gene expression | 16 | 7.6x10^-4^ |
| mRNA decay | GO:1900153 | Deadenylation-dependent mRNA decay | 6 | 9.6x10^-2^ |
| NF-kB signaling | GO:0033256 | I-kappaB/NF-kappaB complex | 5 | 4.8x10^-3^ |
| MAP kinase cascade | GO:0017017 | MAP kinase tyrosine/serine/threonine phosphatase activity | 6 | 6.1x10^-2^ |
| **Down-regulated** |  |  |  |  |
| Metabolisim | GO:0050660 | flavin adenine dinucleotide binding | 16 | 6.8x10^-4^ |
|  | GO:0009055 | electron carrier activity | 19 | 6.8x10^-4^ |
|  | GO:0000062 | fatty-acyl-CoA binding | 10 | 6.9x10^-3^ |
| Endocytosis | GO:0035615 | clathrin adaptor activity | 6 | 2.4x10^-2^ |
|  | GO:0030131 | clathrin adaptor complex | 6 | 4.3x10^-2^ |
|  | GO:0030666 | endocytic vesicle membrane | 11 | 7.4x10^-2^ |
|  | GO:0036020 | endolysosome membrane | 5 | 8.0x10^-2^ |
|  | GO:0030122 | AP-2 adaptor complex | 5 | 8.9x10^-2^ |
